# Engaged parenting, gender, and children's time use in transnational families: An assessment spanning three global regions

**DOI:** 10.1002/psp.2159

**Published:** 2018-05-23

**Authors:** Lucy P. Jordan, Bilisuma Dito, Jenna Nobles, Elspeth Graham

**Affiliations:** ^1^ University of Hong Kong Hong Kong; ^2^ Maastricht University Netherlands; ^3^ University of Wisconsin Madison WI USA; ^4^ University of St. Andrews St. Andrews UK

**Keywords:** children's time use, migrant parenting, transnational families

## Abstract

Global circuits of migration regularly separate parents from children. How families navigate this separation has changed markedly. The sharp decline in the cost of international communication makes possible new forms of transnational parenting. In many contexts, migrants are now actively engaged parents, involved in decisions, knowledgeable of children's schooling, employment, and activities, and in some cases, even conversant face‐to‐face with children via videoconferencing. These practices, however, are not universal. We use data from surveys in three countries to document the frequency and variability of intensive, engaged transnational parenting in the diverse global regions of Asia, Africa, and the Americas. We then ask whether the organisation of children's lives—specifically, time allocated to school homework, leisure, and household chores—varies by the degree to which migrant parents stay connected to sending homes. The gender of the migrant parent, stay‐behind caregiver, and the gender of the child emerge as explanatory factors for engaged parenting and children's time use. However, and unexpectedly, in the Philippines, migrant mothers are less likely to practice engaged parenting. In sending households, girls in two of the three countries spend more time doing household chores than boys, but parental migration does not mitigate this difference. Although we find some evidence of more traditional gender practices, we also find exceptions that suggest potentially fruitful avenues for future research.

## 1 INTRODUCTION

Global circuits of migration regularly separate parents from children, and these separations may last for years. Two decades ago, such separations defined a stark division of labour in families: caregivers in sending regions raised nonmigrant children; migrants earned money and, when possible, remitted resources to sending households. More recently, the sharp decline in the cost of international communication and the attendant spread of communication technology has made a different type of family organisation possible (Baldassar, Nedelcu, Merla, & Wilding, 2016; Boehm, 2001). Globally, many migrants are now actively engaged parents, involved in daily parenting decisions, knowledgeable of children's schooling, employment, and activities, and increasingly, even conversant with children via videoconferencing.

These experiences are far from universal, and whether, when, and why migrant parents stay intensively connected with sending households remains relatively underexamined. Although economic resources likely shape patterns of engagement, well‐resourced migrants are not equally engaged in sending community life. Other barriers to communication for migrants exist, including those from employment responsibilities and new domestic responsibilities, and vary widely across origin and destination countries. Further, gendered expectations and norms around how mothers and fathers should be involved in parenting, as well as the gender of the stay‐behind child, may influence the practice of transnational parenting.

The implications of a shift towards connected transnational parenting—for union longevity, migrant, and children's welfare alike—promises to be an important avenue of research (Graham, Jordan, & Yeoh, 2015; Nobles, 2011). Decades of scholarship on families divided by other processes (e.g., divorce, separation, and deployment) have emphasised the value of maintained connections for family outcomes, particularly when the relationship between the separated parent and child/children's caregiver is amicable (Amato, 2000; Carlson, 2006).

One of the primary motivations for parental migration is to enhance their children's life chances and schoolwork is a major topic of conversation when parents contact children (Asis & Ruiz‐Marave, 2013). Money earned abroad often pays for better, or additional, schooling, and the general expectation is that children in transnational families will apply themselves to their studies both in and after school. However, parental migration may also require a child to contribute more to the household to cover the tasks previously undertaken by the migrant parent, putting pressure on the time available for study. This reallocation may be gendered, as when daughters are required to take on housework or the care of younger siblings in the absence of their migrant mother. Not only is the way that children's time is distributed across different activities likely to vary by child age and household, but it may also vary by how actively involved migrant parents are in the everyday lives of their children. There is currently very little empirical evidence on the time use of children in transnational families.

This study contributes to addressing this gap by investigating the gendered dimensions of migrants' parenting and children's time use in diverse global regions. First, we use data from sample surveys in three different countries to document the frequency and variability of intensive, engaged transnational parenting, and whether this varies by migrant gender. We then ask whether time allocated to school homework, leisure, and household chores—varies by the degree to which migrant parents stay connected to sending homes. We define engaged migrant parenting as occurring when migrants have a high remittance intensity (contribute financial remittances frequently or, for Mexico, to a degree that covers most of children's expenses) and communicate with children in sending homes at least weekly. We maintain that understanding variability in migrants' opportunities to—and choices to—adopt the role of engaged parent has much to tell us about the implications of transnational family arrangements. By comparing transnational families in Southeast Asia, sub‐Saharan Africa, and Latin America, we aim to provide further insight into the diversity of arrangements for “parenting from a distance” (Ambrosini, 2015) and its associations with time use among nonmigrant children.

## 2 TRANSNATIONAL PARENTING AND CHILDREN'S TIME USE: CONCEPTUALISATION AND VARIABILITY

The study of families separated by borders has grown substantially, shifting debates about what constitutes “doing family.” Scholars of transnational families have questioned the presumption that physical proximity is necessary for the maintenance of familial ties (Baldassar et al., 2016; Suárez‐Orozco & Páez, 2008), capturing diversity in the practices of presence and intimacy within transnational families (Baldassar, 2008; Brownlie, 2011; Diminescu, 2008). We build on these studies in defining parental engagement to reflect two key aspects of transnational parenting: providing material resources through remittances and participating in daily life through contact.

Significant changes in interpersonal communication technologies (ICTs) over the past decade (Chib, Wilkin, & Hua, 2013; Madianou & Miller, 2011) make it possible for migrant parents to be actively involved in their children's lives. Contact has thus become an important dimension of “doing transnational family.” Financial and social engagements (contact) are often related in transnational family practices (Levitt & Jaworsky, 2007; Mahler, 2001) and, in contrast to the more negative effects of other types of family separation such as divorce, are more likely to be sustained over time (Nobles, 2011).

To date, more migration scholarship is devoted to remittance behaviour than to communication and active parenting, partially in response to the central role of remittances in theoretical models of migration as a family economic project (Lucas & Stark, 1985; Sana & Massey, 2005). The destination context is a primary predictor of remittance behaviour, largely because of its impact on migrant's labour market opportunities, and the possibilities it offers for the regularisation and integration of migrants (Carling, 2008). For example, among Mexican migrants, education, income, and documentation status are all positively correlated with remittance frequency and monetary value (Goldring, 2004; Valentine, Barham, Gitter, & Nobles, 2017). Generally, the remittance amounts sent by migrant mothers are less than the amounts sent by migrant fathers, probably as a result of women's more limited labour market opportunities and lower earnings. Nevertheless, despite being structurally disadvantaged, women often remit a larger share of their income (Abrego, 2009).

Remittance sending is typically defined within the gendered expectations of migrant parenting. Mothers' feelings of guilt may influence their remitting, even at the cost of their own essential needs (Basa, Harcourt, & Zarro, 2011; Schmalzbauer, 2004). Similarly, remittance sending may be considered fundamental by fathers as it is normatively tied to male breadwinning roles (Dreby, 2006). When fathers fail to remit, expectations (of both parents) are unmet and this can lead to transnational family dissolution (Dreby, 2010; Haour‐Knipe, 2011).

Characteristics of children and their caregivers may also influence whether and how frequently remittances are received, although Nobles (2011) found no evidence that the age and gender of nonmigrant children in Mexico influenced the financial contributions of migrant fathers. Others have emphasised that gender bias may be more evident in the allocation of remittances (Antman, 2012; Bouoiyour & Miftah, 2016). In some contexts, a bias in favour of girls and younger boys has been found in the spending of remittances on education (Acosta, 2006) and health care (Lopez‐Ekra, Aghazarm, Kötter, & Mollard, 2011), whereas other studies have found the opposite effect, with a bias in favour of boys (Hu, 2012; Lu & Treiman, 2007). Some evidence suggests that the relationship between the caregiver and the migrant parent shapes remittances. Divorce, for example, has been found to negatively affect migrant parent–nonmigrant child relationships and also influence the flow and use of remittances (Dreby, 2007).

Knowledge of how nonfinancial aspects of child rearing are accomplished from a distance is mostly based on ethnographic work. Some studies indicate that the lack of face‐to‐face contact constrains parent–child intimacy (Boccagni, 2012; Laurie, 2008). Others stress the role ICTs play in enabling a meaningful relationship between parents and children (Cabanes & Acedera, 2012; Haagsman & Mazzucato, 2014; Peng & Wong, 2013). As separation due to migration is the “new normal” in some settings, advancement in ICT has been central to transnational family relationships, leading to a “de‐demonisation” of distance (Baldassar et al., 2016). However, these influences vary depending on social class, gender, children's age, notions of family access, and skills to use new technologies (Baldassar, 2007; Madianou, 2016; Madianou & Miller, 2011; Parreñas, 2005, 2008).

The current investigation seeks a broader understanding of transnational parenting practices and children's lives. Rather than focusing solely on remittance sending, which captures a single dimension of parental involvement, we combine the provision of financial resources with contact frequency between migrant parents and their nonmigrant children to define engaged parenting. We hypothesise that engaged parents are more likely than other migrant parents to influence the organisation of their children's daily lives. As a co‐resident parent plays an important role in managing their child's weekly routine, an engaged migrant parent may well fulfil this role remotely (Fresnoza‐Flot, 2009).

Only a few studies suggest a link between parental migration and children's time use, with equivocal results. Some find that children of migrant parents spend more time on school homework (Botezat & Pfeiffer, 2014), whereas others find that girls especially spend less time on education. Girls are more likely to substitute household chores for school homework, whereas boys are more likely to substitute leisure activities for homework (Nguyen, 2016; Pörtner, 2016). In Mexico, children spend less time studying in the short‐term after their fathers' departures, but study time is replaced with working for pay and teenage boys experience this shift to a greater extent than younger boys or than girls of any age (Antman, 2012). Engaged parenting may both relax the income constraint that leads families to substitute children's schooling with work and allow migrant parents to encourage children's school performance. We expect that, depending on age and gender, children with engaged migrant parents will spend more time on schoolwork relative to those with less engaged migrant parents.

The effect of parental migration on children's time use will depend in part on the context‐specific organisation of children's lives. In general, children in low‐income countries spend more time in both market and household work compared to children in wealthier countries (Hsin, 2007; Larson & Verma, 1999; Lloyd, Grant, & Ritchie, 2008). Parental migration may relax the income constraints that drive children's labour, but when mothers migrate from countries with strong gender norms, daughters might be expected to take over their household tasks (Asis, 2002), and this may be reinforced with the presence of an engaged migrant parent.

Other potential determinants of children's time use include household size and the characteristics of the child's caregiver (Maralani, 2008; Steelman, Powell, Werum, & Carter, 2002). Younger children with more siblings may receive less supervision from a co‐resident parent or caregiver and thus spend more time on leisure. On the other hand, the absence of a parent may increase the importance of peer friendships for nonmigrant older children and thus lead to an increase in time spent with friends. Moreover, greater material resources (e.g., from remittances) may positively influence time spent in leisure (Larson & Verma, 1999). If remittances allow children in sending homes to substitute other activities for employment, whether this is school homework or leisure is likely to be context‐specific.

Migration introduces considerable stress into transnational families, and children's lives are likely to be shaped in important ways by the mental health of nonmigrant caregivers (Graham et al., 2015; Jordan & Graham, 2012; Waldfogel, Craigie, & Brooks‐Gunn, 2010). To our knowledge, no previous studies have examined how adult mental health influences children's time use specifically, but related research provides some insights. Children of parents in mental distress may use hobbies/socialising to reduce stress, whereas worries about financial resources could increase the likelihood of children working for pay or increase motivation to excel in education for future economic security (Bee, Berzins, Calam, Pryjmachuk, & Abel, 2013).

The current study builds on this multidisciplinary literature to address two main research questions:

1. What are the main determinants of engaged parenting (frequent communication and remittance sending) among migrant parents and does this vary according to the gender of the migrant?
2. Do children whose migrant parents are more engaged allocate time (across household chores, school homework, and leisure) differently to those with less engaged migrant parents, and how does this vary by child gender?

As Wu and Cebotari (2018) point out, there remains a need to recognise the complexity of children's experiences in the context of parental migration. By comparing three global sending regions representing diverse cultural contexts, we provide insight into the commonalities and differences of transnational family practices, and their associations with children's daily allocation of time.

## 3 DATA AND METHODS

Conducting a comparative study across different contexts presents a number of challenges (Mazzucato & Dito, 2018). One critical challenge is operationalizing the conceptual domains across unique data sets to allow cross‐country comparison. We use the same measures when possible, with a few exceptions due to data uniqueness (see Appendix S2 for bivariate distributions: “engaged parenting” by each measurement domain for each study country). Further, although each dataset contains the most detailed information available for the study of transnational families, they have different sampling strategies and coverage that impose constraints on comparative analysis. The implications for interpretation and relevant sensitivity tests are considered below.

**Figure 1:**
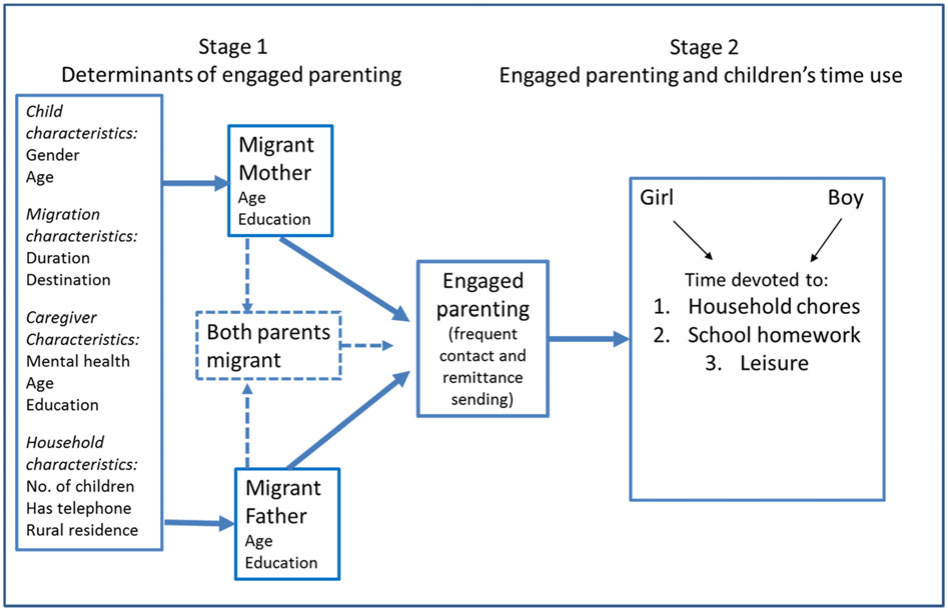
Conceptual framework for the two‐stage analysis

Figure 1 summarises the two‐stage research design and the domains of interest. The rest of this section details the design and discusses the data sources, measures, and modelling strategy.

### 3.1 Data sources

We draw data from three studies on transnational families. Children's Health and Migrant Parents in Southeast Asia (CHAMPSEA) is a longitudinal mixed‐methods study that first conducted interviews with children and other household members in four countries (Indonesia, the Philippines, Thailand, and Vietnam) in 2008–2009. The current analyses use data for the Philippines. The Transnational Child Raising Arrangement between Africa and Europe (TCRAf‐EU) survey data include a school‐based survey with data collected in 2010/2011 in Angola, Ghana, and Nigeria. The current analyses use data for Nigeria. The Mexican Family Life Survey (MxFLS) is a longitudinal household survey in Mexico collected between 2002 and 2010. Table 1 summarises selected characteristics of these surveys for the subsamples included in the current study.

**Table 1:** Summary characteristics of three datasets and the analytical samples

Name	Date	Country	Survey base	Child ages	Interviews	No. of children with migrant parents
[S1]	2008/2009; [2016/17^a^]	Philippines	Household‐based	9–11 years	Child; Caregiver; Responsible adult	244 children (1 index child per household)
[S2]	2011	Nigeria	School‐based	10–14 years	Child	211 children (1 child per household)
[S3]	2002; 2005/06; 2009/13	Mexico	Household‐based	9–14 years	All household members	247 pooled child observations (multiple children per household)

a Ongoing follow‐up in Indonesia and the Philippines data not yet available for analysis.

NB: Sampling designs are accounted for in all descriptive and multivariate analyses. See Section 3 for further details.

Each survey employed a different sampling strategy. In CHAMPSEA, eligible households were either (a) transnational (one or both parents working overseas) or (b) nonmigrant (both parents usually resident at the same address as the index child) for at least 6 months prior to interview. Sampling followed a three‐stage design, with flexible quotas defined by household migration status, child gender, and child age for two groups of children aged 3, 4, and 5, and 9, 10, and 11. The samples include approximately 1,000 households in each country and one index child per household. They are not nationally representative, but, due to tightly specified protocols, they are replicable (see Graham & Yeoh, 2013, for further details). For the current subsample, 65% of children had migrant fathers, 24% had migrant mothers, and 11% had both parents migrant. In the Nigerian TCRAf‐EU data, the school‐based sample from 25 selected schools was stratified by school quality (public/private and junior/senior secondary). One classroom from different grades was included and purposive sampling ensured a sufficient number of children with migrant parents (see Mazzucato et al., 2015, for further details). The dataset, although not nationally representative, contains information on a total of 2,168 children. For the subsample used here, children with migrant fathers (58%) are more common than those with migrant mothers (12%) and those with both parents migrating (29%).

The multilevel MxFLS survey collected data on all members of 8,300 households in 150 communities in Mexico in 2002. Sampling used a nationally representative frame from the 2000 Census. Follow‐up surveys tracked migrants and split‐off households, conducting interviews with new household members. Approximately 90% of original households were located and surveyed in the second wave (2005/2005) and third wave (2009/2013) of the survey (Rubalcava & Teruel, 2013). Both transnational and nonmigrant households were interviewed in each wave; 3% to 5% of children had a parent living in the United States during each interview wave. As international migration of mothers is less common in Mexico (under 2% of households surveyed) and the MxFLS collected detailed data on nonresident parenting by fathers, our analyses focus on migrant fathers.

For the current study, we use CHAMPSEA data collected in 2008 for 244 children aged 9–11 living in transnational households in two provinces in the Philippines. We use the TCRAf‐EU data for 213 junior and senior secondary students, aged 10–14, with internationally migrant parents and living in two major urban areas with high international migration rates in Nigeria. From the MxFLS, we combine 153 observations from the 2005 survey and 94 observations from the 2009/2013 survey of Mexican children aged 9–14 with migrant parents (*N* = 247). We appropriately adjust the standard errors for nonindependence of pooled data in the Mexican sample. All three surveys collected detailed demographic, and socio‐economic for children and their households from which comparable measures of engaged parenting, its determinants and children's time use are derived.

### 3.2 Method

The analysis proceeds in two stages. For the study sites in which multiple children from a household are included, parenting inputs from migrants are recorded for each child individually.

We begin by examining the prevalence of engaged parenting among migrants whose children reside in each study country. We define engaged parents as those who call children at least weekly (for all three studies) and who practice high remittance intensity, either sending remittances “frequently/regularly” 1In CHAMPSEA, frequently/regularly is defined as three or more times in the past 6 months; in the Nigerian TCRAf‐EU data, it is defined as 1 = once a month and several times a year, 0 = once a year and “do not know.” (for CHAMPSEA and TCRAf‐EU or in amounts that “cover most of children's expenses” (for S3). In each of the three study sites, the sending household reports this information. Among 9‐ to 11‐year‐olds (CHAMPSEA) and 9‐ to 10‐year‐olds (MxFLS), the child's primary caregiver reports this information. Among 10‐ to 14‐year‐olds (TCRAf‐EU) and 11‐ to 14‐year‐olds (MxFLS), the child reports the information. We recognise that the difference in who reports may influence the results (see Jordan & Graham, 2012). Single‐year age controls are used to adjust for systematic variation in reporting.

To investigate the main determinants of engaged parenting (Stage 1), we assess within‐ and across‐population variation in engaged parenting among mother and father migrants. We regress the dichotomous engaged parenting indicator on a set of child, migration, caregiver, and sending household‐specific characteristics (Figure 1). The measures were selected based on prior literature and common availability across the three surveys for the key measurement domains (see Appendix S1). These include child age years and gender (male/female), migrant parent's duration of absence by the time of the survey (<12, 12–36, 36+ months), migrant parent's age (39 years or less, 40+ years), migrant parent's level of education (any formal schooling to completed upper secondary, completed upper secondary, or higher), primary caregiver's relation to the child (parent, grandparent, and other kin), caregiver's age (14–39 years, 40+ years), caregiver's level of education (none, any formal schooling to completed upper secondary, completed upper secondary, or higher), caregiver's self‐assessed mental health, 2In CHAMPSEA, mental health is measured using the Self Reporting Questionnaire20 (SRQ‐20) with scores ranging from 0 to 20. A dichotomous measure using the validated cut‐point of 7/8 indicates 1 for presence of problem symptomology (Tuan, Harpham, & Huong, 2004) In the MxFLS, mental health is measured using a scale of depressive symptoms, validated by the Mexican Institute for Psychiatry (Calderón, 1997). The scale ranges from 20 to 80 with higher values indicating worse symptomology. number of children in the sending household, whether the sending household has a landline telephone (yes/no), and whether the household is living in a rural area.

We maximise the similarity of these covariates across study sites. Some marginal variation is required. For example, the ages of parents vary across the sample populations and the country‐specific measures reflect the differences in these distributions. Where migrants' age and education are highly correlated with caregiver's age and education, migrants' age and education are excluded from the main analysis of the Mexican data (but tested in sensitivity analysis). Population density is included for Mexico but not for the Philippines and Nigeria where sampling took place in more urban areas. Notably, for all three countries, the analysis omits a measure of the sending household's wealth or income. As the outcome measure of engaged parenting includes remittance sending, which is closely associated with household wealth, including the latter as an independent variable raises issues of endogeneity. We use migrant's education as a socio‐economic indicator in the study samples because, in the great majority of cases, schooling completion preceded the migration decision.

To address the second research question (Stage 2), we assess whether children of engaged migrant parent(s) experience differences in daily time allocation fitting OLS regression models. Time allocation data were collected separately for each child. We categorise children's time as allocated to (a) household chores (cooking, cleaning, care of younger siblings/older relatives, agricultural labour, getting water or firewood, and helping younger siblings with homework); (b) school homework; and (c) leisure (playing, participating in clubs or other activities, watching TV, and reading). In CHAMPSEA and the MxFLS, these allocations are measured in hours/minutes, for a typical day (CHAMPSEA) or during the week prior to interview (MxFLS). Time spent on household chores is also measured in hours/minutes in the (TCRAf‐EU) data whereas time allocations on school homework and leisure are measured in categorical units (less than an hour per day, 1–2 hr, 2–3 hr, 3–4 hr, 4–5 hr, and more than 5 hr). To enhance comparability and retain the greatest possible level of detail, we assign midpoint minutes to the outcomes in (TCRAf‐EU). Doing so assumes that the distribution of minutes within the category is either uniform, normal, or otherwise has a mean of the midpoint. We have no reason to believe time use follows alternative distributions.

We regress time use on the indicator of engaged parenting adjusting for the controls described above. In study sites capturing families with mothers or fathers (or both) absent, we introduce interactions between the engaged parenting indicator and whether or not the migrant is the child's mother or father. We include each of the covariates described in the first part of the analysis above, with the same site‐specific variations. All the regressions use weights and clustering appropriate to each study's sampling scheme. The MxFLS estimates address the nonindependence among children who contribute an observation in both 2005/2006 and 2009/2013 with a Huber–White cluster estimator. The CHAMPSEA and the TCRAf‐EU Nigerian data analyses account for the occurrence of multiple instances of dyadic data when a child has both parents migrating, taking into account common variance within these households.

Full study sample bivariate descriptive statistics are available in online Appendices. The following section summarises the key findings for the two research questions, describing each country separately. In the Section 5, we then focus on the cross‐cutting theme of gender and explore the comparative dimension of the analyses more fully.

## 4 RESULTS

### 4.1 The prevalence of engaged parenting

**Figure 2:**
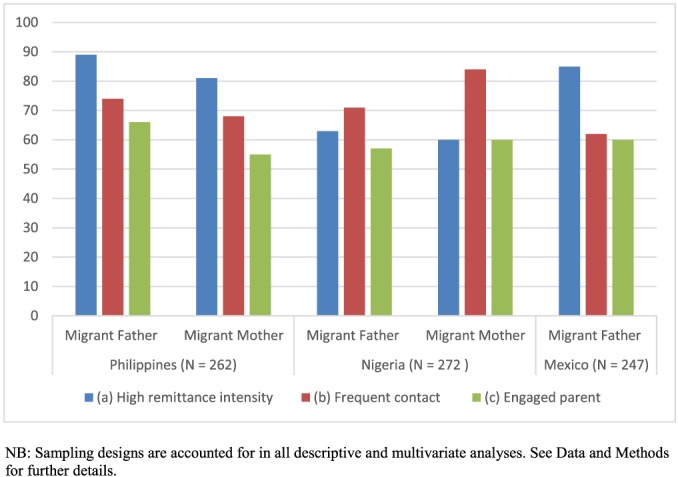
Percentages of migrant fathers and mothers who (a) send frequent/sufficient remittances, (b) contact frequently, and (c) are engaged parents

Figure 2 shows the percentages of engaged migrant mothers and fathers in each sample, along with the percentages for each of the two components of the engaged parenting indicator—high remittance intensity (frequent/sufficient) and frequent contact.

In the Philippines, remittance intensity differs by parent's gender, with fathers more likely to remit frequently compared to mothers. For Nigeria, remittance intensity operates in a similar manner, with more migrant fathers than migrant mothers remitting frequently, although the gender gap is less pronounced. Among Mexican migrant fathers, remittance intensity is high overall (85% send sufficient remittances), and higher than for Nigerian migrant fathers, but lower than for migrant fathers from the Philippines. The remittance intensity of fathers across the study countries echoes the findings of DeWaard et al. (2018), drawing further attention to the need to better understand structural determinants of migrant practices (see also Eremenko & Gonzales, 2018) including remittances.

Regular contact between migrant parents and the sending household also varies by parents' gender, but in the opposite manner for the Philippines and Nigeria. Filipino migrant fathers are more likely than migrant mothers to have weekly or more contact with the sending household, whereas for Nigeria, over 80% of migrant mothers contact their children frequently compared to 71% of migrant fathers. The prevalence of frequent contact among migrant fathers in the Mexican sample is lower than for any parental group from the other study countries.

Additional discordance is evident in migrant parents living abroad identified as “engaged parents”: those with both high remittance intensity and frequent contact with their children in sending households. In the Philippines and Nigerian samples, the percentages of engaged parents are less common than either singular component, indicating that some migrant parents can provide one of these inputs but not both. Mexico is the only case in which engaged parenting appears to be limited largely by contact frequency.

We now turn to our two research questions. Table 2a, 2b, 2c reports results for four multivariate regression models for each country, with respectively (a) engaged parent, and time spent on (b) school homework, (c) leisure, and (d) household chores as the outcome of interest.

**Table 2a:** Regression models for the Philippines

Philippines		Migrant parent	Child		
Outcome:		Engaged parent	Minutes per day school homework (logged)	Minutes per day leisure (logged)	Minutes per day household chores (logged)
Estimation		Logistic	OLS	OLS	OLS
Covariates		[Odds Ratios]	[β]	[β]	[β]
		(1)`	(2)	(3)	(4)
**Migrant parent(s) actively engaged in transnational parenting**			0.054	−0.002	−0.029
			[−0.222–0.330]	[−0.218–0.214]	[−0.472–0.414]
**x Migrant is a mother**			0.304	−0.016	1.267***
			[−0.211–0.820]	[−0.420–0.387]	[0.440–2.094]
**x Migrant is both parents**			−0.334	−0.295	−1.202**
			[−1.068–0.399]	[−0.869–0.280]	[−2.379 to −0.025]
**Child**					
Female		0.964	0.163	−0.053	0.782***
		[0.558 ‐ 1.665]	[−0.048–0.374]	[−0.218–0.112]	[0.444–1.120]
Age	9 years (omitted)				
	10 years	0.614	0.086	−0.166*	0.036
	11 years	[0.330–1.144]	[−0.161–0.334]	[−0.360–0.028]	[−0.362–0.434]
		1.243	0.072	0.116	−0.089
**Migration**		[0.590–2.616]	[−0.206–0.350]	[−0.102–0.333]	[−0.535–0.358]
Duration	12 months or less (omitted)				
	13–36 months	1.042	−0.159	0.123	−0.218
		[0.438–2.475]	[−0.502–0.184]	[−0.145–0.392]	[−0.769–0.332]
	36 months +	1.241	−0.263	0.162	−0.344
		[0.535–2.877]	[−0.593–0.066]	[−0.096–0.420]	[−0.873–0.185]
Destination	Middle East (omitted)				
	Asia	1.986	−0.02	−0.014	−0.457
		[0.763–5.169]	[−0.390–0.349]	[−0.303–0.276]	[−1.051–0.136]
	Seafaring	0.388	0.377	0.19	−0.514
		[0.093–1.628]	[−0.214–0.969]	[−0.273–0.653]	[−1.463–0.435]
	Other	1.854	0.426***	0.027	−0.177
		[0.825–4.166]	[0.124–0.729]	[−0.210–0.264]	[−0.662–0.309]
**Migrant**					
Father (omitted)					
Mother		0.432**	−0.192	0.035	−0.616*
		[0.203–0.922]	[−0.605–0.221]	[−0.288–0.359]	[−1.279–0.047]
Both parents migrating		0.284*	0.702**	0.089	0.491
		[0.070–1.153]	[0.018–1.387]	[−0.447–0.625]	[−0.608–1.590]
Age	25–39 years (omitted)				
	40–54 years	0.915	−0.105	−0.141	−0.18
		[0.470–1.780]	[−0.358–0.149]	[−0.339–0.058]	[−0.586–0.227]
Education	None through completed upper secondary (omitted)				
	Completed upper secondary or higher	1.354	−0.055	−0.132	−0.097
		[0.594–3.089]	[−0.388–0.278]	[−0.393–0.129]	[−0.632–0.438]
**Primary caregiver**					
Relationship to child					
Parent (omitted)					
Grandparent		1.882	−0.302	−0.005	−0.778**
		[0.571–6.202]	[−0.749–0.146]	[−0.355–0.345]	[−1.496 to −0.060]
Other kin/non‐kin caregiver		4.023**	0.076	0.248	−1.440***
		[0.955–16.949]	[−0.414–0.565]	[−0.135–0.632]	[−2.227 to −0.654]
Age	25–39 years (omitted)				
	40–54+ years	0.937	0.164	0.197*	0.195
		[0.467–1.882]	[−0.103–0.432]	[−0.012–0.407]	[−0.234–0.625]
Education	None (omitted)				
	Any formal schooling < completed upper secondary	0.782	0.368	−0.163	−0.124
		[0.245–2.495]	[−0.085–0.820]	[−0.517–0.192]	[−0.850–0.603]
	Completed upper secondary or higher	1.499	0.387*	−0.021	−0.372
		[0.506–4.440]	[−0.032–0.806]	[−0.349–0.307]	[−1.045–0.300]
Mental health		0.6	−0.064	0.012	−0.083
		[0.295–1.221]	[−0.356–0.228]	[−0.217–0.241]	[−0.552–0.386]
Sending household					
Number of children		0.923	−0.032	−0.026	0.015
		[0.725–1.173]	[−0.127–0.064]	[−0.101–0.049]	[−0.139–0.168]
Telephone in household		3.043	−0.004	−0.641	1.914**
		[0.158–58.716]	[−1.094–1.086]	[−1.495–0.213]	[0.163–3.664]
Constant			3.533***	5.767***	1.106
			[4.762–6.772]	[4.762–6.772]	[−0.955–3.167]
Chi‐sq		23.96			
BIC			794.1012	665.9937	1042.188
N		244	244	244	244

* *p* < .1;

** *p* < .05;

*** *p* < .01.

**Table 2b:** Regression models for Nigeria

		Migrant parent	Child		
Outcome		Engaged parent	Minutes per day school homework (logged)	Minutes per day leisure (logged)	Minutes per day household chores (logged)
Estimation		Logistic	OLS	OLS	OLS
Covariates		[Odds Ratios]	[β]	[β]	[β]
		(1)`	(2)	(3)	(4)
**Migrant parent(s) actively engaged in transnational parenting**			−0.201	0.581	−0.0756
			[−0.577–0.174]	[−0.149–1.311]	[−0.901–0.750]
**x Migrant is a mother**			−0.204	−1.047	−0.357
			[−1.079–0.671]	[−2.747–0.654]	[−2.280–1.566]
**x Migrant is both parents**			0.497	−0.0330	−0.371
			[−0.113–1.106]	[−1.218–1.152]	[−1.711–0.969]
**Child**					
Female		0.74	−0.190	−0.516**	0.251
		[0.399–1.373]	[−0.455–0.0754]	[−1.031 to −0.001]	[−0.332–0.834]
					
Age	10 years (Omitted)				
	11 years	2.212	0.374	−0.400	0.866
		[0.650–7.530]	[−0.164–0.912]	[−1.446–0.645]	[−0.317–2.049]
	12 years	2.223	0.501*	−0.433	0.229
		[0.695–7.112]	[−0.0143–1.016]	[−1.435–0.568]	[−0.904–1.362]
	13 years	3.053*	0.519**	−0.0386	0.801
		[0.957–9.737]	[0.0144–1.024]	[−1.020–0.942]	[−0.309–1.911]
	14 years	3.397**	0.406	−0.0883	0.466
		[1.086–10.619]	[−0.102–0.913]	[−1.075–0.898]	[−0.649–1.582]
**Migration**					
Duration	6 months or less (omitted)				
	36 months +	2.628***	0.0627	0.110	0.177
		[1.384–4.988]	[−0.218–0.343]	[−0.435–0.655]	[−0.439–0.794]
**Migrant**					
Father (omitted)					
Mother		0.332	0.581	1.206	1.158
		[0.063–1.746]	[−0.267–1.429]	[−0.442–2.855]	[−0.706–3.023]
Both parents migrating		1.524	0.354	0.567	0.407
		[0.628–3.701]	[−0.213–0.920]	[−0.533–1.668]	[−0.838–1.651]
Age	39 years or less (omitted)				
	40+ years	1.425	0.312	0.978**	−0.691
		[0.473–4.291]	[−0.175–0.800]	[0.0306–1.925]	[−1.762–0.380]
Missing		0.818	0.360	0.975*	−0.855
		[0.238–2.813]	[−0.179–0.899]	[−0.0727–2.024]	[−2.040–0.331]
Education	None through completed upper secondary (omitted)				
	Some (completed) university	0.265	0.0404	−0.635	0.111
		[0.045–1.571]	[−0.628–0.709]	[−1.934–0.665]	[−1.359–1.582]
Missing		0.356	−0.112	−0.454	0.162
		[0.055–2.311]	[−0.838–0.615]	[−1.866–0.958]	[−1.434–1.759]
**Primary caregiver**					
Female		0.428	0.269	0.373	0.616
		[0.120–1.530]	[−0.228–0.766]	[−0.593–1.339]	[−0.477–1.708]
Education	None through completed upper secondary (omitted)				
	Some (completed) university	1.098	0.0778	−0.187	0.143
		[0.497–2.425]	[−0.257–0.413]	[−0.838–0.464]	[−0.593–0.880]
**Sending household**					
Number of children		0.863*	−0.137***	−0.108	−0.144*
		[0.736–1.012]	[−0.206 to −0.0682]	[−0.241–0.0261]	[−0.296–0.00681]
Household has landline telephone		1.304	0.323**	0.531**	−0.383
		[0.692–2.458]	[0.0531–0.593]	[0.00713–1.056]	[−0.976–0.210]
Constant			3.733***	3.164***	2.771**
			[2.596–4.869]	[0.955–5.374]	[0.272–5.270]
BIC			651.72	930.89	982.61
Chi‐sq		22.12			
N		210	210	210	210

* *p* < .1;

** *p* < .05;

*** *p* < .01.

**Table 2c:** Regression models for Mexico

		Migrant parent	Child		
Outcome		Engaged parent	Minutes per day school homework (logged)	Minutes per day leisure (logged)	Minutes per day household chores (logged)
Estimation		Logistic	OLS	OLS	OLS
Covariates		[Odds Ratios]	[β]	[β]	[β]
		(1)`	(2)	(3)	(4)
**Migrant parent(s) actively engaged in transnational parenting**					
**x Child is a girl**			0.305	0.332	−0.73
			[−0.110–0.719]	[−0.273–0.937]	[−1.584–0.124]
**x Child is a boy**			0.418	−0.121	0.315
			[−0.070–0.907]	[−0.461–0.218]	[−0.615–1.244]
**Child**					
Female		2.156	0.198	−0.341	1.561**
		[0.976 ‐ 4.762]	[−0.304–0.699]	[−1.072–0.390]	[0.571–2.550]
Age:	9 years (omitted)				
	10 years	1.504	−0.617	0.008	0.745
		[0.390–5.800]	[−1.303–0.069]	[−0.712–0.728]	[−0.433–1.923]
	11 years	2.933	−0.402	0.249	0.86
		[0.791–10.875]	[−0.912–0.108]	[−0.266–0.764]	[−0.209–1.929]
	12 years	1.332	0.31	0.082	0.619
		[0.371–4.787]	[−0.176–0.796]	[−0.413–0.577]	[−0.454–1.693]
	13 years	1.455	0.011	0.187	1.375*
		[0.414–5.118]	[−0.525–0.546]	[−0.328–0.703]	[0.244–2.505]*
	14 years	1.793	−0.247	0.03	1.936***
		[0.448–7.167]	[−0.858–0.364]	[−0.518–0.577]	[0.876–2.997]
**Migration**					
Duration	less than 12 months (omitted)				
					
	12–36 months	0.482	0.517	−0.286	−0.285
		[0.140–1.657]	[−0.145–1.179]	[−0.764–0.192]	[−1.255–0.685]
	36+ months	0.635	0.481	−0.048	−0.17
		[0.211–1.915]	[−0.091–1.053]	[−0.335–0.239]	[−1.026–0.687]
**Primary caregiver (child's mother)**					
					
Age:	28–39 years (omitted)				
	40+ years	3.592**	−0.071	0.078	−0.386
		[1.473–8.758]	[−0.443–0.300]	[−0.199–0.355]	[−1.167–0.395]
Education	None (omitted)				
	Any formal schooling < completed upper secondary	2.166	0.243	0.114	0.276
		[0.714–6.568]	[−0.313–0.798]	[−0.279–0.508]	[−0.695–1.248]
	Completed upper secondary or higher	6.121*	0.054	−0.19	0.591
		[1.229–30.478]*	[−1.152–1.259]	[−0.755–0.374]	[−1.098–2.279]
Mental health (Index 20–80)		0.948*	0.011	0.009	0.033
		[0.908–0.989]*	[−0.012–0.033]	[−0.007–0.025]	[−0.007–0.073]
					
**Sending household**					
Number of children		0.973	0.001	−0.022	0.049
		[0.715–1.324]	[−0.137–0.140]	[−0.119–0.076]	[−0.194–0.291]
Has a telephone		1.846	0.015	−0.134	0.079
		[0.812–4.198]	[−0.281–0.311]	[−0.480–0.211]	[−0.555–0.712]
Rural area		2.516*	−0.102	−0.241	0.241
		[1.119–5.657]	[−0.364–0.160]	[−0.498–0.016]	[−0.370–0.852]
Constant			2.707***	5.355***	0.338
			[1.701–3.713]	[4.549–6.161]	[−1.299–1.975]
BIC			737.86	679.26	1066.88
Chi‐sq		28.06			
N		247	247	247	247

* *p* < .1;

** *p* < .05;

*** *p* < .01.

### 4.2 The determinants of engaged parenting

First, odds ratios (ORs) are estimated by regressing the outcome engaged parenting on a series of predictors for each sending household. The key determinants of engaged parenting are gendered, although in the case of Mexico, the effects are indirect via the primary caregiver. For the Philippines, migrant mothers are less likely to be engaged parents than migrant fathers, and parents are less likely to be engaged when both are migrants. The likelihood of engaged parenting may be influenced by alternative care arrangements. In the majority of sending households in the Philippines sample, it is the nonmigrant parent who is the child's caregiver. However, when mothers migrate, there is a greater chance of a nonparental caregiver taking on this role (12% compared to only 2% when the father migrates). We find that when the child is being cared for by someone other than the stay‐behind parent or grandparent, migrant parents are more likely to be engaged (OR = 4.023, *p* < .01). The only other direct migration determinant is for the Nigerian sample where migrants away for longer are more likely to be engaged parents (OR = 2.628, *p* < .01). In the case of the Philippines, there is no significant contribution from other migration characteristics, such as country of destination.

The characteristics of the caregiver (most likely to be the mother), including age, mental health, and education, are important determinants of engaged parenting among Mexican migrant fathers. Measures of caregiver's mental health are generated from different survey instruments, and thus, the estimate magnitudes cannot meaningfully be compared. Estimate direction is relevant, however, with poorer mental health indicated by higher values. We therefore expect to see an inverse relationship between mental health among nonmigrant caregivers and migrant parent engagement with children in sending households because, at least in some cases, caregivers act as “gatekeepers” who facilitate interaction between children and absent parents. If the child's caregiver suffers from anxiety and depression, then their facilitating role may not be fulfilled. Alternatively, the causal direction may be reversed if caregiver mental health worsens when a migrant parent is not in regular contact. The results in Table 2aa–c (Model 1 in each) show that the estimate is negative (OR < 1) for both the Philippines and Mexico samples, but only in the model for Mexico is it statistically significant (Table 2ac, Model 1, OR = 0.948; *p* < .01).

Given known financial barriers to communication, and the likelihood that better educated migrants typically have stronger earning potential, engaged parenting might be associated with completed schooling of both the migrant parent and the child's caregiver. We do not find strong support for this. The pattern only appears in Mexico, where migrant father engaged parenting is positively associated with increased levels of nonmigrant mothers' educational attainment (see Table 2ac, Model 1). Separate tests (not shown) using migrant fathers' education in the Mexican sample indicate the same pattern.

Household and child characteristics are not universally important determinants, although, in the Nigerian case, child age is positively associated with engaged parenting (Age 13: OR = 3.053, *p* < .10; Age 14: OR = 3.397, *p* < .01), whereas the number of children in the household (OR = 0.863, *p* < .10) is negatively associated with the outcome.

Across all included variables and the three countries, patterns are inconsistent. Nevertheless, these results suggest that the genders of the migrant parent and selected caregiver characteristics are important determinants of engaged parenting, though not always in the expected direction. In addition to caregiver (maternal) education, caregiver age and household geography also help to explain variation in transnational engaged parenting in Mexico. Mexican children with caregivers (mothers) who are older and located in more rural areas are more likely to receive engaged migrant fathering (see Table 2ac, Model 1).

### 4.3 Engaged parenting and children's time use

Stage two considers the relationship between engaged parenting and children's time allocation. There are a variety of ways in which engaged parenting might influence the amount of time children spend on these activities, as well as a gender dimension given that expectations for boys and girls are likely to differ.

### 4.4 School homework

Model 2 (in each country's table) shows no direct effect of engaged parenting across the three surveys on children's time spent on school homework. Nevertheless, there is a direct migration effect when both parents are migrant in the Philippines sample, with more time allocated to homework (Table 2aa, Model 3, β = 0.702, *p* < .01). There is also an indirect migration effect in the Philippines, whereby children whose parents are working overseas in less common destinations are significantly more likely to spend time doing school homework compared to children whose parents are working in the Middle East, Asia, or seafaring (Table 2aa, Model 3, β = 0.426, *p* < .001).

Further, child and household characteristics contribute to understanding time allocated to homework in the Nigerian sample, with older children more likely to spend time doing homework but children with more siblings less likely to spend time on homework. There is no observable gender effect for child, migrant, or caregiver in the time children devote to homework across the three study countries.

### 4.5 Leisure

Model 3 (in each country's table) shows no significant associations between children's time spent in leisure and migrants' engaged parenting across the three samples. In the Philippines and Mexico samples, we find little evidence of a relationship with any of our key predictors. Nor does there appear to be a socio‐economic gradient in leisure activities. We might expect that an offsetting income effect of remittance sending would reduce children's need to engage in household chores, freeing up time for activities including leisure, whereas frequent contact may be expected to increase time spent on homework, relative to television watching, for example. When we analyse remittance sending and frequent contact separately, neither is associated with leisure for children in any of the three countries (not shown). The results for the Nigerian sample contribute most to understanding differences in time spent by children in leisure activities; child characteristics (age of the child), as well as household socio‐economic status (presence of landline telephone), are positively associated with time spent in leisure although girls are less likely than boys to devote time to leisure. The age of migrant parents (Nigeria) and of caregivers (Philippines) are also positive determinants of leisure time.

### 4.6 Household chores

Model 4 reports that child gender is an important dimension of time spent on household chores for two of the three samples. For both the Philippines and Mexico, we find a gendered effect whereby girls are significantly more likely to spend time undertaking household chores compared to boys. The gendered effect is further accentuated in the Philippines sample, with migrant gender and the caregiver's relationship to the child also being important predictors. Children whose mothers are engaged migrant mothers, along with children with both parents migrating and engaged, are less likely to spend time doing household chores. Further, children with nonparental caregivers (grandparents or other kin/nonkin) are less likely to spend time doing household chores. In the case of Nigeria, more children in the household, the only significant predictor, decreases a child's time spent on household chores.

## 5 DISCUSSION AND CONCLUSION

Dramatic changes in communication technologies resulting in diverse methods for engagement and reduced costs are offering new opportunities for transnational families to maintain presence across great distances. The current study brings together data from three different surveys of migration and family life representing major sending regions of global migrants in Asia, Africa, and Latin America. To advance understanding of diversity in transnational parenting, we address two related research questions on the determinants and implications of engaged parenting. The findings provide evidence about transnational family organisation, gendered transnational parenting, and the influence of engaged parenting on children's daily routines, highlighting avenues for further scholarly enquiry.

Our first question examines the main determinants of engaged parenting with a particular focus on migrant parent gender. The parenting measure combines remittance intensity with frequent communication between migrant and origin household. One noticeable conclusion is the absence of significant associations between engaged parenting and many variables often related to well‐being in transnational families. We had expected to observe relationships with explanatory domains such as migration and household characteristics. The characteristics of migration, including work destinations and documentation status, can influence the opportunities migrants have to engage with the household of origin (Constable, 2013). However, we found little evidence that migration experiences influence parenting practices in the Philippines and Nigeria, where this variation is measured. Neither did we find substantial evidence for the influence of household socio‐economic status on migrant parents' engagement, with the exception of Mexico where parental education is a key determinant of children's receipt of engaged parenting. Because authorisation status and attendant employment conditions are correlated with education among Mexican migrants (e.g., Massey & Riosmena, 2010), variability in parental schooling may well pick up migrants' time and money to invest in children in sending households.

Gender (of the migrant parent and of the child's caregiver) is the key explanatory factor for engaged parenting. However, and unexpectedly, in the case of the Philippines, it is migrant mothers who are less likely to practice engaged parenting. To some extent, this provides contrary evidence to what Eremenko and Bennett (2018) posit regarding a lack of engagement by migrant fathers. It further contrasts with the findings of previous, mainly ethnographic, studies on gender and the use of ICT among migrant groups (Cabanes & Acedera, 2012; Chib et al., 2013). We observe this result first in our bivariate analyses (Figure 2), and then in the multivariate analyses for the determinants of engaged parenting (Table 2aa–c, Model 1). In our sample, Filipina migrant mothers are taking a less active role in parenting from a distance than some scholarship suggests (Madianou & Miller, 2011). This might be due to structural barriers reducing the opportunities for migrant mothers to contact their families back home, but further examination of occupational type and documentation status failed to support this suggestion (results not shown). As Eremenko and Gonzalez (2018) demonstrate, structural factors of destination and origin contexts are influential determinants of transnational family dynamics. Given current dataset limitations, we are not able to conduct a more detailed analysis of the possible meaning and influence of financial constraints on migrant mothers' contact patterns in this study.

It is also possible that the relationship between the migrant parent and the stay‐behind caregiver influences engaged parenting. Perhaps when the child is in the primary care of the co‐resident father, the migrant mother feels more confident about the child's well‐being and therefore feels less need for frequent contact, although Mazzucato et al. (2015) found that Angolan migrant parents in Europe experienced lower emotional well‐being when the caregiver of the child in Angola is the other biological parent. Alternatively, the reverse causal pathway may operate, with qualitative aspects of the relationship between the migrant mother and stay‐behind father inhibiting contact. Maternal migration is sometimes referred to as “Filipino divorce” (Timmerman, Martiniello, Rea, & Wets, 2015) and the marriages of some couples in the sample may be under strain. The available data do not include information on the quality of relationships. Qualitative findings by Manuh (1999) and Schmalzbauer (2004) indicate the tensions and distrust that couples experience due to separation following international migration, and such tensions could limit engaged parenting. Formal divorce in Mexico has been rare (Frank & Wildsmith, 2005) until recent increases in the last decade (Arias, 2013). All of the couples in the Mexico sample are partnered, because fathers' residence in the United States is reported by partnered mothers (see Nobles, 2011, for details). Thus engaged parenting for the full population of children in sending homes may be overrepresented. It is likely that the small fraction of children living away from migrant and divorced parents receive the least amount of engaged parenting (Dreby, 2010). Conflict within the parental dyad is a strong predictor of nonresident–parent and child interaction in most research and warrants future study in transnational families. To explore this with our current data, we estimated models for the Nigeria and Philippines samples excluding “both parents away,” and found no substantive differences. Overall, our findings add to the debates raised by Caarls et al. (2018) and DeWaard et al. (2018) regarding the importance of studying couple and gendered migrations, and the greater salience of the care triangle for understanding children's well‐being in the context of migration (Jordan & Graham, 2012).

Our second research question considers the relationship between engaged migrant parenting and the organisation of children's daily lives, as well as variability by child gender. We posited that actively engaged migrant parents would shape children's time use in the three domains of school homework, leisure activities, and household chores. The theoretical rational for investigating these domains is based on the limited literature about children's time use in less economically advanced countries (Hsin, 2007; Larson & Verma, 1999) and the few studies that have examined time use and parental migration (Nguyen, 2016; Pörtner, 2016). In societies with gendered norms about the division of labour within families, time spent doing household chores may be greater among girls (Nguyen, 2016). Indeed, in line with previous research, we find evidence that girls in two of the three countries spend more time doing household chores than boys. Only in the Philippines is there an unexpected relationship between engaged parenting and time spent doing household chores. The effect is gendered—but not in the anticipated direction. Children of engaged migrant mothers are less likely to spend time doing household chores. Children in the Philippines sample are younger than the majority of those in the other two studies and age may play a role here. Alternatively, schoolwork may be given priority over household chores as Filipina migrant mothers encourage their children to study hard (Asis & Ruiz‐Marave, 2013). These findings may also be indicative of the transmission of social remittances, where alternative conceptualizations of gendered possibilities are exchanged (Levitt, 1998). Evidence from this study suggests that maternal engagement with the sending household may contribute to the dynamics of time use within the household, but not always in a way that results in female children taking on more household chores in the absence of their migrant mother. In Mexico, girls with migrant fathers take on substantially more household labour than do boys with migrant fathers; nevertheless, this does not appear to differ by whether or not the child's father is an engaged parent. Neither do remittances appear to be allocated to reducing household labour among girls relative to boys.

Our examination of time spent on homework did not offer any further insight into the relationship between engaged parenting and children's time use across the three country samples. A priori, we anticipated that engaged parenting would increase children's time spent doing school homework, given the emphasis on children's schooling among migrant families (Asis & Ruiz‐Marave, 2013; Dreby & Stutz, 2012) and ethnographic evidence that migrant parents often discuss schoolwork with children (Dreby, 2010), yet we found no supporting evidence. Nevertheless, there are other covariate associations of interest. In both the Philippines and Nigerian samples, children with both parents migrant are most likely to spend time doing school homework. Although there is not a specific caregiver effect, this finding suggests that alternative caregiving arrangements, as well as who migrates, may play a role. It could be that both parents migrating is accompanied by higher expectations for children's schooling and/or that children respond to the “sacrifice” of family life made by their mother and father by studying harder to please absent parents. Additionally, in the Philippines, children whose migrant parents are in less common destinations are more likely to spend more time doing school homework. These destinations account for about 18% of the total sample and include places in Europe and North America. Such destinations could be associated with increased financial security for migrants because of higher earning potential. These settings also offer higher returns to human capital that make educational qualifications more desirable. Both features may influence expectations about time spent by children doing homework. Larson and Verma's (1999) meta‐analysis of children's time use indicates a clear relationship between increasing household and community socio‐economic status and the time children spend on school homework. The influence of having two migrant parents could operate similarly, although this association could reflect the greater propensity of alternative caregivers to encourage children to spend more time doing homework, perhaps to demonstrate respect for the wishes of migrant parents to support their children's education.

Overall, our study offers a range of insights into the practices of transnational families within three global regions of significant international out‐migration. It is not, however, without limitations. We have already noted the limitations imposed by the lack of comparable financial data that could allow greater specification of household wealth. An added challenge to comparability comes from the different sampling designs and content of the three surveys, as well as child versus adult reporting on key measures, which could influence the findings. In particular, the school‐based Nigerian survey raises issues of children's knowledge about their parents' remitting behaviour. Another notable limitation is the lack of precise measures for comparison (e.g., on migration and caregiver characteristics including caregiver mental health). Finally, the data used in this study were collected between 2008 and 2010. Since then, the cost of communication technology has decreased further whereas simultaneously the methods of ICT have increased significantly. How this has shaped the entry into and maintenance of transnational parenting will be an important avenue for future research. Our assessment of existing scholarship, along with the results presented here, suggests that some barriers to staying connected to children in sending households will not be easily overcome by reductions in communication costs. Nevertheless, it is possible that the prevalence rates of “engaged parenting” presented in this study underestimate rates observed today.

A strength of the study is that analysis across different global settings promises a better understanding of commonality and difference in transnational family practices as global circuits of migration become an increasingly important feature of contemporary life. Many of the other authors in this issue conduct comparative analyses within regions (e.g., DeWaard et al., 2018 in Latin America and Caarls et al., 2018 in Africa) whereas our study stretches the limits of comparability across three global regions of Asia, Latin America, and Africa. This ambitious comparative investigation of engaged parenting and its relationship to children's time use offers insight into the salience of gender within transnational families. Several findings suggest fruitful avenues for future research. For example, more detailed information about household economics and the opportunities for engaging in communication and exchange of financial resources could provide a basis for extending understanding of why mothers are less likely than fathers to be “engaged parents.” Further, longitudinal data would allow more detailed analysis of how the financial costs associated with migration, including debts incurred in the migration process, impact on the ability—or choice—of migrant parents to send remittances to, and contact, their families back home. Longitudinal studies that examine the sequential changes in household gender roles, including parenting and caregiving, could offer deeper insight into prevalent practices within contemporary transnational families.

## FUNDING

Funding support for this study is from Singapore Ministry of Education Academic Research Fund Tier 2 (MOE2015‐T2‐1‐008); Hong Kong Research Grants Council through its General Research Fund (Project 17606815); Wellcome Trust UK (GR079946/B/06/Z and GR079946/Z/06/Z).

## Supporting information

Supporting Information S1
Appendix S1 Conceptual Measurement Domains and Country Specific Measures
Appendix S2: Table S1A: Means and percentages for the Philippines
Appendix S2: Table S1B: Means and percentages for Nigeria
Appendix S2: Table S1C: Means and percentages for Mexico
