# Supplementary material for: Engaged parenting, gender, and children's time use in transnational families: An assessment spanning three global regions
Source: Popul Space Place. 2018 May 23;24(7):e2159. doi: 10.1002/psp.2159 (PMC6473664; doi:10.1002/psp.2159)
Supplement: Supplementary file 1 — Supporting Information S1 Appendix S1 Conceptual Measurement Domains and Country Specific Measures Appendix S2: Table S1A: Means and percentages for the Philippines Appendix S2: Table S1B: Means and percentages for Nigeria Appendix S2: Table S1C: Means and percentages for Mexico [file PSP-24-na-s001.docx]

APPENDICES

| Appendix A: Conceptual Measurement Domains and Country Specific Measures | | | |  |  |
| --- | --- | --- | --- | --- | --- |
|  | Mexico | | Nigeria | | Philippines |
| **Time use measures** |  | |  | |  |
| Minutes per day housework | X | | X | | X |
| Minutes per day homework | X | | Categorical | | X |
| Minutes per day leisure/play | X | | Categorical | | X |
| **Child** |  | |  | |  |
| Female | X | | X | | X |
| Age |  | |  | |  |
| 9-11 years |  | |  | | X |
| 9-14 years | X | |  | |  |
| 10-14 years |  | | X | |  |
| **Migration** |  | |  | |  |
| Duration |  | |  | |  |
| 12months or less (omitted category) | X | |  | | X |
| 13-36 months | X | |  | | X |
| 36months + | X | | X | | X |
| Destination |  | |  | |  |
| Middle East (Omitted Category) |  | |  | | X |
| Asia |  | |  | | X |
| Seafaring |  | |  | | X |
| Other |  | |  | | X |
| **Migrant** |  | |  | |  |
| Father (omitted category) | X | | X | | X |
| Mother |  | | X | | X |
| Both parents migrating |  | | X | | X |
| Age |  | |  | |  |
| 39 years or less (omitted category) | X | | X | | X |
| 40+ years | X | | X | | X |
| Education |  | |  | |  |
| Any formal schooling < completed upper secondary |  | | X | | X |
| Completed upper secondary or higher |  | | X | | X |
| **Primary caregiver** |  | |  | |  |
| Relationship to child |  | |  | |  |
| Parent (omitted category) | X | |  | | X |
| Grandparent |  | |  | | X |
| Other relation or non-kin |  | |  | | X |
| Gender of caregiver |  | | X | |  |
| Age |  | |  | |  |
| 14-39 years (omitted category) | X | |  | | X |
| 40+ years | X | |  | | X |
| Education None (omitted category) |  | | X | | X |
| Any formal schooling < completed  upper secondary |  | | X | | X |
| Completed upper secondary or higher |  | | X | | X |
| Mental health | X | |  | | X |
| **Sending household** |  | |  | |  |
| Number of children | X | | X | | X |
| Has a telephone | X | | X | | X |
| Rural area | X | |  | |  |
| N | 247 | | 211 | | 244 |
| X = Dataset has this component | |  | |  |  |

II. Bivariate by Engaged Parenting by Survey A/B/C

| Appendix B: Table 1A: Means and percentages for the Philippines |  |  |  |  |  |  |
| --- | --- | --- | --- | --- | --- | --- |
|  | Engaged Parent | | |  | All |  |
|  | YES |  | NO |  |  |  |
|  | Mean / % | (SD) | Mean / % | (SD) | Mean / % | (SD) |
| **Time use measures** |  |  |  |  |  |  |
| Minutes per day housework | 26.13 | (28.45) | 20.76 | (21.05) | 24.13 | (26.03) |
| Minutes per day homework | 63.88 | (42.66) | 56.11 | (37.83) | 60.98 | (41.03) |
| Minutes per day leisure/play | 186.26 | (109.56) | 181.87 | (110.42) | 184.62 | (109.69) |
|  |  |  |  |  |  |  |
| **Child** |  |  |  |  |  |  |
| Female | 49.70 |  | 47.96 |  | 49.05 |  |
|  |  |  |  |  |  |  |
| Age 9 years (omitted) | 38.18 |  | 33.67 |  | 36.50 |  |
| 10 years | 33.33 |  | 45.92 |  | 38.02 |  |
| 11 years | 28.48 |  | 20.41 |  | 25.48 |  |
|  |  |  |  |  |  |  |
| **Migration** |  |  |  |  |  |  |
| Duration |  |  |  |  |  |  |
| 12months or less (omitted category) | 12.73 |  | 14.29 |  | 13.31 |  |
| 13-36 months | 29.70 |  | 33.67 |  | 31.18 |  |
| 36months + | 57.58 |  | 52.04 |  | 55.51 |  |
|  |  |  |  |  |  |  |
| Destination |  |  |  |  |  |  |
| Middle East (Omitted Category) | 58.79 |  | 65.31 |  | 61.22 |  |
| Asia | 12.73 |  | 12.24 | Asia | 12.55 | Asia |
| Seafaring | 2.42 |  | 5.10 |  | 3.42 |  |
| Other | 26.06 |  | 17.35 | Other | 22.81 | Other |
|  |  |  |  |  |  |  |
| **Migrant** |  |  |  |  |  |  |
| Father (omitted category) | 67.88 |  | 59.18 |  | 64.64 |  |
| Mother | 21.82 |  | 28.57 |  | 24.33 |  |
| Both parents migrating | 10.30 |  | 12.24 |  | 11.03 |  |
|  |  |  |  |  |  |  |
| Age |  |  |  |  |  |  |
| 25-39 years (omitted category) | 57.58 |  | 56.12 |  | 57.03 |  |
| 40-54+ years | 42.42 |  | 43.88 |  | 42.97 |  |
|  |  |  |  |  |  |  |
| Education |  |  |  |  |  |  |
| Any formal schooling <upper secondary (omitted) | 10.30 |  | 18.37 |  | 13.31 |  |
| Completed upper secondary or higher | 89.70 |  | 81.63 |  | 86.69 |  |
|  |  |  |  |  |  |  |
| **Primary caretaker** |  |  |  |  |  |  |
| Relationship to child |  |  |  |  |  |  |
| Parent (omitted category) | 75.76 |  | 77.55 |  | 76.43 |  |
| Grandparent | 14.55 |  | 17.35 |  | 15.59 |  |
| Other relation or nonkin | 9.70 |  | 5.10 |  | 7.98 |  |
|  |  |  |  |  |  |  |
| Age |  |  |  |  |  |  |
| 14-39 years (omitted category) | 50.91 |  | 47.42 |  | 49.62 |  |
| 40+ years | 49.09 |  | 52.58 |  | 50.38 |  |
|  |  |  |  |  |  |  |
| Education None (omitted category) | 9.70 |  | 9.18 |  | 9.51 |  |
| Any formal schooling < completed upper secondary | 15.76 |  | 28.57 |  | 20.53 |  |
| Completed upper secondary or higher | 74.55 |  | 62.24 |  | 69.96 |  |
|  |  |  |  |  |  |  |
| Mental health | 13.33 |  | 22.45 |  | 16.73 |  |
|  |  |  |  |  |  |  |
| **Sending household** |  |  |  |  |  |  |
| Number of children | 2.69 | (1.15) | 2.85 | (1.22) | 2.75 | (1.175) |
|  |  |  |  |  |  |  |
| Has a telephone | 99.39 |  | 97.96 |  | 98.86 |  |
|  |  |  |  |  |  |  |
| N | 165 |  | 97 |  | 262 |  |

| Appendix B: Table 1B: Means and percentages for Nigeria | Engaged Parent | | |  | All |  |
| --- | --- | --- | --- | --- | --- | --- |
|  | YES |  | NO |  |  |  |
|  | Mean / % | (SD) | Mean / % | (SD) | Mean / % | (SD) |
| **Time use measures** |  |  |  |  |  |  |
| Minutes per day housework on a typical week | 86.17 | (113.97) | 91.24 | (116.06) | 88.29 | (114.66) |
| Minutes per day spent on homework | 167.28 | (99.20) | 174.47 | (105.86) | 170.29 | (101.91) |
| Minutes per day spent on leisure | 128.92 | (104.88) | 125 | (110.60) | 127.28 | (107.13) |
|  |  |  |  |  |  |  |
| **Child** |  |  |  |  |  |  |
| Female | 45.57 |  | 50.88 |  | 47.79 |  |
|  |  |  |  |  |  |  |
| Age (years) 10 years (omitted) |  |  |  |  |  |  |
| 11 years | 13.92 |  | 25.44 |  | 18.75 |  |
| 12 years | 20.25 |  | 20.18 |  | 20.22 |  |
| 13 years | 27.85 |  | 20.18 |  | 24.63 |  |
| 14 years | 32.28 |  | 21.05 |  | 27.57 |  |
|  |  |  |  |  |  |  |
| **Migration** |  |  |  |  |  |  |
| Duration 36+ months | 72.26 |  | 52.38 |  | 64.71 |  |
|  |  |  |  |  |  |  |
| **Migrant** |  |  |  |  |  |  |
| Father (omitted category) |  |  |  |  |  |  |
| Mother | 10.61 |  | 12.37 |  | 12.32 |  |
| Both parents migrating | 33.33 |  | 36.08 |  | 28.91 |  |
|  |  |  |  |  |  |  |
| Age |  |  |  |  |  |  |
| 39 years or less (omitted category) |  |  |  |  |  |  |
| 40+ years | 68.99 |  | 55.26 |  | 63.23 |  |
|  | 22.15 |  | 35.09 |  | 27.57 |  |
| Education |  |  |  |  |  |  |
| None through completed upper secondary (omitted category) |  |  |  |  |  |  |
| Some (completed) university | 77.85 |  | 78.95 |  | 78.31 |  |
|  | 15.82 |  | 18.42 |  | 16.91 |  |
| **Primary caretaker** |  |  |  |  |  |  |
| Female | 74.05 |  | 81.58 |  | 77.21 |  |
|  |  |  |  |  |  |  |
| Education |  |  |  |  |  |  |
| None through completed upper secondary (omitted category) |  |  |  |  |  |  |
| Some (completed) university | 78.67 |  | 83.02 |  | 80.47 |  |
|  |  |  |  |  |  |  |
| **Sending household** |  |  |  |  |  |  |
| Number of children | 1.04 | (2.16) | 1.51 | (2.44) | 1.24 | (2.28) |
|  |  |  |  |  |  |  |
| Household has landline telephone | 43.67 |  | 44.73 |  | 44.11 |  |
|  |  |  |  |  |  |  |
| N | 158 |  | 114 |  | 211 |  |

| Appendix B: Table 1C: Means and percentages for Mexico |  |  |  |  |  |  |
| --- | --- | --- | --- | --- | --- | --- |
|  | Engaged Parent | | |  | All |  |
|  | YES |  | NO |  |  |  |
|  | Mean / % | (SD) | Mean / % | (SD) | Mean / % | (SD) |
| **Time use measures** |  |  |  |  |  |  |
| Average minutes per day housework in the week prior to survey | 68.31 | (78.93) | 71.12 | (124.57) | 69.64 | (99.91) |
| Average minutes per day homework in the week prior to survey | 60.1 | (40.73) | 51.42 | (39.79) | 55.98 | (40.37) |
| Average minutes per day leisure in the week prior to survey | 250.12 | (138.34) | 262.29 | (149.80) | 255.9 | (142.81) |
|  |  |  |  |  |  |  |
| **Child** |  |  |  |  |  |  |
| Female | 59 |  | 42.5 |  | 51.22 |  |
|  |  |  |  |  |  |  |
| Age: 9 years | 9.85 |  | 16.44 |  | 12.95 |  |
| 10 years | 13.6 |  | 18.58 |  | 16 |  |
| 11 years | 24.88 |  | 11.91 |  | 18.72 |  |
| 12 years | 20.33 |  | 25.12 |  | 22.61 |  |
| 13 years | 15.56 |  | 15.87 |  | 15.7 |  |
| 14 years | 15.78 |  | 12.08 |  | 14.02 |  |
|  |  |  |  |  |  |  |
| **Migration** |  |  |  |  |  |  |
| Duration less than 12 months (omitted category) | 6.5 |  | 9.1 |  | 19.43 |  |
| 12-36 months | 29.1 |  | 32.3 |  | 30.61 |  |
| 36+ months | 49 |  | 59 |  | 49.96 |  |
|  |  |  |  |  |  |  |
| **Primary caretaker (child's mother)** |  |  |  |  |  |  |
|  |  |  |  |  |  |  |
| Age: 28-39 years (omitted category) | 64.4 |  | 81.1 |  | 72.37 |  |
| 40+ years | 35.6 |  | 18.9 |  | 27.63 |  |
|  |  |  |  |  |  |  |
| Education None (omitted category) | 4.7 |  | 8.6 |  | 6.55 |  |
| Any formal schooling < completed upper secondary | 89.8 |  | 88.6 |  | 89.24 |  |
| Completed upper secondary or higher | 5.5 |  | 2.8 |  | 4.21 |  |
|  |  |  |  |  |  |  |
| Mental health (Index 20-80) | 26.7 | (7.96) | 27.91 | (9.88) | 27.27 | (8.79) |
|  |  |  |  |  |  |  |
| **Sending household** |  |  |  |  |  |  |
| Number of children | 3.27 | (1.47) | 3.3 | (1.57) | 3.28 | (1.51) |
|  |  |  |  |  |  |  |
| Has a telephone | 66.43 |  | 53.24 |  | 60.2 |  |
|  |  |  |  |  |  |  |
| Rural area | 53.1 |  | 40.3 |  | 47.08 |  |
|  |  |  |  |  |  |  |
| N | 147 |  | 100 |  | 247 |  |
